# Development and Evaluation of Two-Phase Gel Formulations for Enhanced Delivery of Active Ingredients: Sodium Diclofenac and Camphor

**DOI:** 10.3390/pharmaceutics16030366

**Published:** 2024-03-05

**Authors:** Giedre Kasparaviciene, Yuliia Maslii, Nataliia Herbina, Daiva Kazlauskiene, Mindaugas Marksa, Jurga Bernatoniene

**Affiliations:** 1 Department of Drug Technology and Social Pharmacy, Lithuanian University of Health Sciences, LT-50161 Kaunas, Lithuania; giedre.kasparaviciene@lsmu.lt (G.K.); yuliia.maslii@lsmu.lt (Y.M.); nataliia.herbina@lsmu.lt (N.H.); 2 Department of Industrial Technology of Drugs, National University of Pharmacy, 61002 Kharkiv, Ukraine; 3 Department of Analytical and Toxicological Chemistry, Lithuanian University of Health Sciences, LT-50161 Kaunas, Lithuania; daiva.kazlauskiene@lsmu.lt (D.K.); mindaugas.marksa@lsmu.lt (M.M.); 4 Institute of Pharmaceutical Technologies, Faculty of Pharmacy, Medical Academy, Lithuanian University of Health Sciences, LT-50161 Kaunas, Lithuania

**Keywords:** bigel, sodium diclofenac, camphor, organogel/hydrogel ratio, development, evaluation

## Abstract

The formulation of biphasic gels as potential semi-solid carriers for hydrophilic and lipophilic active substances is promising for the development of pharmaceutical preparations. The aim of this study was to design a stable bigel composition and to determine the influence of the organogel/hydrogel ratio on the gel’s physical-chemical and structural-mechanical properties. The investigated compositions of organogel/hydrogel remained stable at ratios ranging from 5/95 to 40/60. After texture and microstructure analysis, bigels with 20/80 and 25/75 ratios were selected as carriers for the active ingredients, sodium diclofenac and camphor, for use as topical preparations for the treatment of muscle-joint inflammation and pain. Although other researchers have published data on the preparation and evaluation of bigels, there are no scientific results on the development of a two-phase gel with our proposed combination of APIs. Sodium diclofenac release was found to be higher when combined with camphor, which revealed the advantages of the biphasic formulation. The pseudoplastic behavior, thixotropy, and thermal stability of flow of the studied bigel samples was investigated by rheological analysis. Ongoing stability studies confirmed the minimal 6-month period.

## 1. Introduction

The most popular group of medicines for the treatment of muscle-joint pain [1,2,3,4] that occupy a leading place in the world in terms of consumption are the nonsteroidal anti-inflammatory drugs (NSAIDs). This is explained by their high effectiveness in pain syndrome of inflammatory origin and their spectrum of pharmacological effects: anti-inflammatory, analgesic, and antipyretic [5,6]. 

However, systemic use of NSAIDs—injections, oral and rectal administration—can cause complications in the gastrointestinal tract, cardiovascular system, and kidneys [7]. Therefore, local semi-solid forms of NSAIDs for topical application, which have very low risk of systemic side effects, are now attracting attention [1,7,8].

The skin is a powerful barrier to any medication transport. The stratum corneum, consisting of keratin-rich dead cells embedded in a complex lipid matrix containing cholesterol, ceramides, and free fatty acids, prevents the permeation of hydrophilic molecules. In turn, the viable epidermis contains a large amount of water, which stops the diffusion of lipophilic substances [9,10]. Therefore, to ensure the successful transfer of the active substance into the subcutaneous tissue and deeper structures, in pharmaceutical forms for dermal application, it is advisable to combine lipophilic and hydrophilic properties.

One of the most common NSAIDs is diclofenac sodium, which combines anti-inflammatory, analgesic and antirheumatic effects. Diclofenac suppresses both the exudative and proliferative phases of inflammation, which makes it possible to use it during the treatment of many musculoskeletal system diseases. It blocks both isozymes of cyclooxygenase (COX). Inhibition of COX-1 when using diclofenac is less pronounced compared to that occurring with ibuprofen and naproxen therapy, with the result that gastrointestinal tract lesions occur less often. Diclofenac inhibits COX-2 to a lesser extent than celecoxib, etoricoxib, and rofecoxib, which helps reduce the risk of cardiovascular complications. This balanced effect of diclofenac provides a combination of high therapeutic activity and good tolerability of treatment [11]. In addition to inhibiting prostaglandins, diclofenac sodium has been found to inhibit leukocyte migration to the site of inflammation. To some extent, the drug can affect the balance of cytokines, reducing the concentration of interleukin-6 and increasing the content of interleukin-10. This change in the ratio of these substances helps to slow down the secretion of anti-inflammatory factors [12,13]. A reduction in the release of free oxygen radicals, which occurs under the influence of diclofenac sodium, can also contribute to a decrease in the inflammatory process activity [14]. In addition to pronounced anti-inflammatory activity, diclofenac sodium has complex effects on various mechanisms of pain perception, providing effective suppression of pain syndromes of various aetiologies, as well as central and peripheral antinociceptive effects [15,16]. Topical diclofenac may be preferred over oral NSAIDs, especially for older patients and those who have comorbid conditions and/or risk factors for various systemic adverse events associated with oral NSAIDs, particularly when used at high doses or for long durations [17].

The pharmaceutical industry produces diclofenac sodium for local use in various dosage forms: cream (2%); ointment (1%, 2%); gel (1%, 2%, 5%); and transdermal patch (15 mg/day), etc. [18].

It should be noted that medicines with diclofenac sodium for local use are mainly mono-preparations [18]. In addition, the literature indicates that diclofenac has low absorption into the systemic bloodstream when applied locally [19]. It is known that substances that have a local irritant effect and increase local blood flow increase the permeability of the skin when they are included in local medicinal forms. These substances include ethyl alcohol, camphor, menthol, essential oils, and capsaicin [20,21,22]. 

Camphor is a natural compound with a terpene backbone that is often used in topical formulations, including for muscle-joint pain, due to its analgesic, antiseptic, local irritant, and anti-inflammatory properties. Studies have found that camphor causes hyperpolarization of the membranes of nociceptive nerve endings, thus causing their desensitization. It has an exciting effect on cold receptors, which explains the subjective feeling of coolness at the application site of camphor preparations. Exciting sensitive nerve endings of the skin reflexively improves the trophism of organs and tissues [23,24]. Most of the camphor adverse reactions (convulsions, lethargy, ataxia, severe nausea, and vomiting) are caused by gastrointestinal absorption; however, when applied to the skin, even in large quantities, camphor rarely causes systemic poisoning [25].

The 3% to 11% concentration of camphor has been approved by the FDA for topical use as a pain reliever and anaesthetic [26]. The pharmaceutical industry produces medicines with a concentration of camphor in the range from 0.5% to 10.8%, although in some cases, higher concentrations are used [27,28]. The most common camphor drugs are camphor alcohol (10%), oil solutions (10%, 20%), liniment “Camphocin” (15%), camphor ointment (10%), and gel “Camphotrol^®^” (PureTek Corporation, Panorama City, CA, USA) with a combination of 4% camphor and 10% menthol [18]. Lotion with 0.5% camphor and 0.5% menthol; gel with 0.3% camphor, 0.3% menthol and 0.3% phenol [29]; spray with 3% menthol, 4% camphor and 20% methyl salicylate [30]; and ointment with camphor and menthol at 0.025%, 0.05% and 0.1% of each compound [31] are found in the literature. According to published data, the probability of adverse reactions of camphor applied topically, especially at low concentrations, is relatively low [32].

Thus, for higher effectiveness, a combination of diclofenac sodium and camphor as an active pharmaceutical ingredient (APIs) are used for bigel formulation. The use of camphor improves skin permeation, and increases the bioavailability of diclofenac and the analgesic potential of the combined medicine.

**Figure 1:**
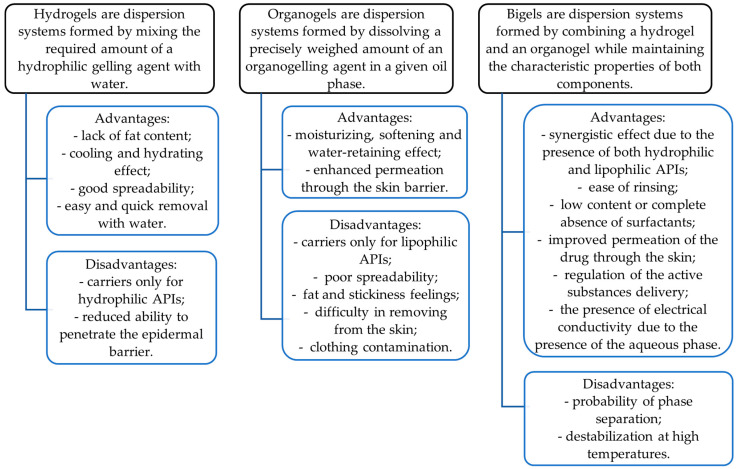
Characteristics of hydrogels, organogels, and bigels as gelified dosage forms.

To date, bigels are a promising dosage form that can accommodate both hydrophilic and lipophilic drugs. They have the advantages of both phases, aqueous (hydrogel) and oily (organogel), and demonstrate better properties than any of the individual gels [33]. Their characteristics are shown in Figure 1. The combination of two gels can demonstrate a synergistic effect, resulting in improved drug permeation due to the presence of both hydrophilic and lipophilic carriers [34,35]. They can easily penetrate the skin and hence are often chosen for topical or transdermal drug delivery [36,37]. 

In a bigel, two gels—an organogel and a hydrogel—are prepared individually using a special gelling agent [35]. A list of different gellificators is presented in Table 1.

Therefore, important aspects of the development of any medicinal product are the selection of effective active pharmaceutical ingredients, rational excipients, and a convenient dosage form to ensure the maximum therapeutic effect. The formulation of biphasic gel as potential semi-solid carrier for hydrophilic and lipophilic active substances is promising for the development of pharmaceutical preparations. 

The aim of our work is to develop the composition and evaluate the quality of a bigel containing a combination of diclofenac sodium and camphor for muscle-joint pain relief.

## 2. Materials and Methods

### 2.1. Materials 

The substances used for the bigel formulation were as follows: diclofenac sodium (Merck KgaA, Darmstadt, Germany), camphor (racemic) (Merck KgaA, Darmstadt, Germany), sodium carboxymethyl cellulose (Merck KgaA, Darmstadt, Germany), stearic acid (Carl Roth GmbH + Co. KG, Karlsruhe, Germany), sorbitan monostearate (Thermo Fisher Scientific GmbH, Dreieich, Germany), olive oil (Lelia Foods S.A., Avlida, Greece), and distilled water (LUHS laboratory, Kaunas, Lithuania). 

The following chemical reagents and standards were used for analysis: 96.3% (*v*/*v*) ethanol (SC Stumbras, Kaunas, Lithuania); acetonitrile (≥99%, Sigma–Aldrich, Taufkirchen, Germany); acetic acid (≥99%, Sigma–Aldrich, Taufkirchen, Germany); methanol (≥99%, Sigma–Aldrich, Taufkirchen, Germany); hexane (≥99%, Sigma–Aldrich, Taufkirchen, Germany). Ultrapure water was purified with a Millipore Water cleaning system (Millipore Corp., Bedford, MA, USA).

### 2.2. Bigel Preparation

The hydrogel was prepared using 5% sodium carboxymethylcellulose (Na-CMC) and distilled water. Na-CMC was poured onto the surface of distilled water heated to a temperature of 60–70 °C. After swelling, the mixture was stirred constantly until a gel system was obtained. The organogel was prepared by heating a mixture of 12% stearic acid, 1% sorbitan monostearate (span 60) and 87% olive oil at 75 ± 2 °C under constant stirring (50 rpm). The hot mixture was cooled to room temperature (25 ± 2 °C), during which, the organogel formed. Bigels were produced by mixing the organogel with the hydrogel in the following ratios: 5/95; 10/90; 15/85; 20/80; 25/75; 30/70; 35/65; 40/60; 45/55; and 50/50. The organogel was incorporated into the hydrogel by stirring with a digital mixer IKA^®^ EUROSTAR 200 digital (IKA, Toledo, OH, USA) at room temperature. A mixing speed of 500 rpm was used until a homogeneous system was formed (~5 min). Sodium diclofenac was incorporated into the hydrogel base at a 1.0% rate and camphor was incorporated at a 0.5% rate into the organogel base. After mixing, the bigels were stored in well-sealed containers at room temperature until analysis.

### 2.3. Evaluation of Sensory Parameters

The sensory parameters—colour, odour, and homogeneity (lack of separation)—were analysed by applying the sample (organogel, hydrogel, bigels) to a glass slide in a 2–4 mm layer.

### 2.4. Microscopic Analysis of the Bigels

The particles of the oil phase of the produced bigels were observed with an optical microscope Motic^®^ BA310 Ltd. (Motic Europe, Xiamen, China). The images were magnified 100 times and the Motic^®^ Images Plus program calculated the size of selected oil droplets. For each bigel, 10 random droplets of oil were selected, and their average diameter was determined.

### 2.5. Particle Size and Distribution Measurements

Oil particle size and its distribution were assessed using a Mastersizer 3000 with a Hydro EV unit (Malvern Panalytical Ltd., Malvern, UK). Bigel samples without dilution were added dropwise to the dispersant (water) to obtain laser obscuration between 9.5% and 10.5%. The pump speed was kept constant at 2400 rpm. The refractive indexes used for the dispersant and dispersing material were 1.330 and 1.467, respectively. The particle size distribution was measured in five runs and the average was calculated. The formulations were described by the percentile (D10, D50, and D90) values.

### 2.6. Determination of the Bigels’ Stability

A centrifugation test was used to determine the initial stability of the two-phase system. Test samples (1 ± 0.5 g) were placed in special centrifuge tubes (Eppendorf, Hamburg, Germany) and centrifuged in a high-speed centrifuge, Sigma 3–18 KS (Sigma Laborzentrifugen, Berlin, Germany). The test was performed at a temperature of 25 ± 2 °C, at a speed of 3000 rpm, and with a test time of 5 min. The stability of the system was assessed by visual observation of the separation of the oil and water phases. 

The freeze–thaw test consisted of 24 h of freezing (−20 ± 2 °C) followed by 12 h of thawing (25 ± 2 °C). Freeze–thaw cycles were repeated 3 times, and at the end of each cycle, a visual evaluation was performed. If a physical phase separation was observed the formulation was no longer investigated. 

The centrifugation and freeze–thaw tests were performed for freshly prepared samples and after 1, 3, and 6 months of storage. 

### 2.7. Viscometric Evaluation

The viscosity of the organogel, hydrogel and bigels was measured with a Fungilab Alpha Series R Model Rotational Viscometer (Fungilab, Barcelona, Spain). Equal amounts (50 ± 0.01 g) of the samples were analysed with an L4 spindle at 12 rpm shear rate. The measurements were performed at room temperature.

### 2.8. Determination of pH Values

Solutions of 5% concentration of the organogel, hydrogel and bigels in distilled water were analysed. All the mixtures were sonicated for 5 min until the gel sample mixed with the water. The mixture was then filtered through a paper filter. The pH value was measured with a Winlab Data Line pH meter (Windaus. Labortechnik, Clausthal-Zellerfeld, Germany). Three replicate analyses were performed at room temperature for each sample.

### 2.9. Texture Analysis

The mechanical properties of the organogel, hydrogel and bigels were determined using a TA.XT.plus (Stable Micro Systems Ltd., Godalming, Surrey, UK) texture analyzer. The back extrusion and spreadability tests were performed. All the tests were conducted at room temperature and repeated three times.

#### 2.9.1. Back Extrusion Test 

**Figure 2:**
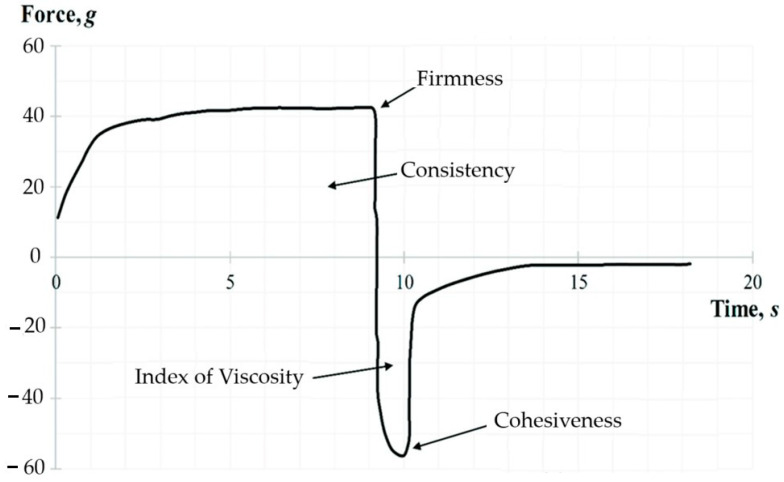
A representative graph of the back extrusion test.

The back extrusion test was performed using the Back Extrusion Rig A/BE. The sample was placed in a standard container and was compressed by a disk (40 mm in diameter) which penetrated to a depth of 25 mm at the rate of 1 mm/s. As a result, a plot of force versus time was obtained for each sample using the Texture Expert for Windows v. 1.05 software (Stable Micro Systems, Haslemere, UK). The following parameters were determined: firmness (g), consistency (g·s), index of viscosity (g·s), and cohesiveness (g). A representative graph of the back extrusion test force is shown in Figure 2. The positive part of the graph demonstrates the downward movement of the probe. The maximum compressing force required for the bigel’s deformation represents the firmness parameter. While the negative peak demonstrates the force required to break the intermolecular bonds—the cohesiveness of the gel. The area above the graph represents the consistency, and the area under the graph the index of viscosity.

#### 2.9.2. Spreadability Test

**Figure 3:**
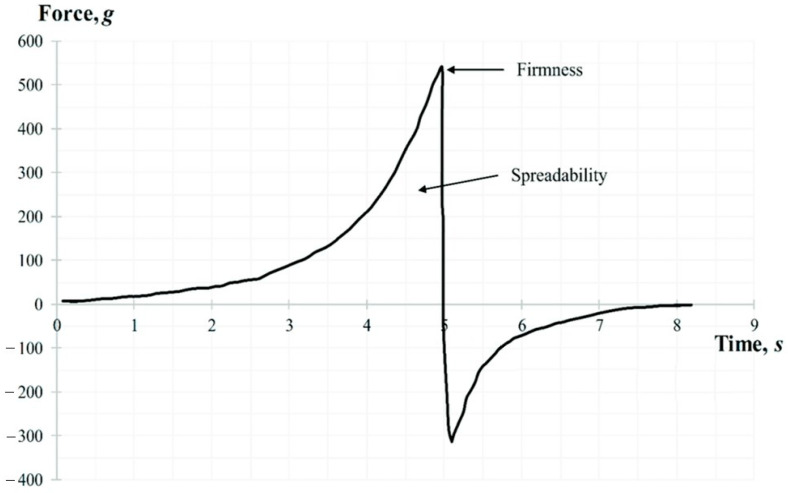
A representative graph of the spreadability test.

The spreadability test was performed using the TTC Spreadability Rig HDP/SR. The sample was placed in a standard container and compressed by conical probe which penetrated to a depth of 17 mm at the rate of 3 mm/s. The following parameters were determined: firmness (g), and spreadability (g·s). A representative graph of the spreadability test force is shown in Figure 3. While the probe moves down, the gel flows outward between the surfaces of the cone-shaped container and the probe. The area above zero represents the spreadability of the sample. The peak of the positive part of the graph shows the gels ability to flow, its firmness.

### 2.10. Rheological Studies of the Bigels

The rheoparameters were evaluated using a rotational rheometer MCR102 (Anton Paar BmbH, Graz, Austria) equipped with a plate–plate geometry. The temperature was controlled with a Peltier system. The bigel sample (about 2.0 g) was placed in the space between the two flat plates with a gap of 1.0 mm. The rheoparameters were measured by rotating the upper plate at different shear rates—from 0.01 s^−1^ to 100 s^−1^—and in the opposite direction at temperatures of 25 ± 0.1 °C and 32 ± 0.1 °C. An assessment of the flow of the biphasic systems was also performed by studying the consistency coefficient and the flow behavior index at different temperatures.

### 2.11. In Vitro Release Test

A USP 4 apparatus (Sotax CE7 smart with Sotax CP 7–35 pump, Sotax AG, Aesch, Switzerland) was used for in vitro release testing. Flow through cells (22.6 mm diameter) with an adapter for semi-solid formulations were used in a closed system. The system was temperature-controlled at 32 ± 0.5 °C. Approximately 1 ± 0.1 g of the tested formulation was placed into the adapter and fitted with a cellulose membrane (Sigma Aldrich, St. Louis, MO, USA). Then, 50 mL of distilled water was circulated at a flow rate of 16 mL/min (120 pulse/min). Samples of 1.5 mL were withdrawn and replaced with fresh media at specified time intervals (1, 2, 3, 4, 5, and 6 h). The studies were repeated thrice for each bigel sample. All the samples were analysed by HPLC. The cumulative percentage of released sodium diclofenac was calculated and is discussed in the results section.

### 2.12. Quantification of Diclofenac by High-Pressure Liquid Chromatography–Photodiode Array Analysis

**Figure 4:**
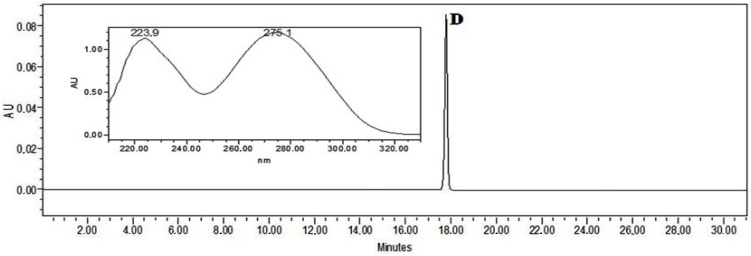
The chromatogram of sodium diclofenac standard solution (D—sodium diclofenac).

Quantitative analysis of diclofenac was carried out on a Waters Alliance 2695 liquid chromatograph equipped with a Waters 996 photodiode array detector (PDA) and an ACE C18 (250 mm × 4.6 mm id), particle size 5 μm column (Advanced Chromatography Technologies, Aberdeen, Scotland). The mobile phase consisted of solvents A (acetic acid (0.5%)) and B (acetonitrile). The linear gradient elution profile was as follows: 95% A/5% B at 0–5 min, 40% A/60% B at 10 min, 39% A/61% B at 12 min, 38% A/62% B at 14 min, 37% A/63% B at 15 min, 35% A/65% B at 16 min, 30% A/70% B at 20 min, 5% A/95% B at 25–30 min, and 95% A/5% B at 31 min. The flow rate was 1 mL/min, and the injection volume was 10 μL. Ultraviolet absorption was measured in the range from 200 to 300 nm. Detection of diclofenac was performed using 0.1 mg/mL of methanolic diclofenac standard solution (Figure 4). The retention time (RT) was on average 17.79 min. Sample preparation: 0.5 g of the bigel was weighed and diluted in 100 mL of methanol by using an ultrasound bath for 10 min. This solution was used for analysis by HPLC (Figure 4).

A 0.1 mg/mL sodium diclofenac methanolic solution was assigned as a marker that could be used to evaluate the quality and authenticity of sodium diclofenac in the bigel. The confirmation of the chromatographic peak identity was achieved by comparing the retention time and spectral characteristics (*λ* = 200–300 nm) of the eluting peak with the reference compound. The stock solution of the reference standard was prepared by dissolving it in methanol. A calibration curve was established by diluting the stock solution with methanol in appropriate quantities. The quantities of sodium diclofenac were calculated from an external standard calibration curve established on five concentrations in the following range: 0.5–0.03125 mg/mL. Each sample was analysed in triplicate and the mean value was used for calculation. The concentration of sodium diclofenac was expressed as mg/mL, then converted to percentage concentration. The analytical characteristics of the HPLC method validation data are shown in Table 2.

### 2.13. Quantification of Camphor by Gas Chromatography–Mass Spectrometry Headspace Analysis 

Gas chromatography–mass spectrometry headspace analysis was performed on a GCMS-QP2010 Ultra system (Shimadzu, Tokyo, Japan). An 800 mg bigel sample was dissolved in 1 mL of hexane (≥99%, Sigma–Aldrich, Germany) and 35 mg of pure camphor (Merck KgaA, Darmstadt, Germany) was dissolved in 1 mL of hexane (≥99%, Sigma–Aldrich, Germany) as a standard solution. The vial was sealed with an aluminium crimp cap with a PTFE/silicon septum (Millipore Sigma, St. Louis, MO, USA). The sealed vials were mixed thoroughly prior to being placed on the headspace auto-sampler (AOC-5000 Plus, Shimadzu, Tokyo, Japan) for injection. The column used was a 30 m × 0.25 mm i.d. × 0.25 9 of 12 µL film thickness RTX-5MS. The column oven temperature was 30 °C, and injection temperature was 250 °C. A split injection mode was used, and the split ratio was 1:100. The flow control mode pressure was 45.0 kPa, and the total flow was 103.1 mL/min. The flow rate of the helium (99.999%, AGA, Vilnius, Lithuania) carrier gas was set at 0.99 mL/min. The linear velocity was 35.7 cm/s, and the purge flow was 3.0 mL/min. The oven temperature was maintained at 30 °C for 3 min after injection and then programmed at 2 °C/min to 150 °C, at which level the column was maintained for 30 min. The mass spectrometer ionization mode was electron ionization, and the detector gain was 1.47 kV. The scan ACQ mode was used, and the event time was 0.20 s. The speed of the scan was 1428 amv/s. The mass scan range was 29.00–300.00 *m*/*z*.

### 2.14. Statistical Analysis

The mean values and standard deviations of the results were calculated using Microsoft Office Excel 2016 (Redmond, WA, USA). The significance of differences was evaluated using Students *t*-test. The differences were statistically significant at *p* < 0.05.

## 3. Results and Discussion

### 3.1. Evaluation of Physical and Mechanical Properties of Organogel and Hydrogel

Development of pharmaceutical products is influenced by complex quantity and quality factors: APIs, excipients, quality, and stability of the product. The therapeutic effect of semi-solid formulations is based on the functionality of the active ingredient’s carrier. The bigel formulation was designed from the initial composition of the excipients and focused on system stability, viscosity, and mechanical properties evaluation. Topical dosage forms should be homogeneous, have a good spreadability on the skin and be easily removed from it, without leaving a greasy and sticky feeling. Therefore, textural and rheological characteristics, which can affect the mechanical and sensorial properties of the product, are important quality parameters [34,64]. In addition, bigels, in order not to damage the protective skin barrier, must have a pH that corresponds to the physiological pH of the skin—4–6.5 [65]. 

The constituents of the bigel—hydrogel and organogel—were evaluated first. The physical properties, including the pH and viscosity, were evaluated. During the texture analysis, the mechanical properties were determined: firmness, spreadability, consistency, cohesion, and index of viscosity (Table 3).

According to the results given in Table 3, the pH values of both gels were similar, very close to neutral media. This indicates that the samples may not cause any irritation to the skin and hence can be used for topical applications. The viscosity of the sodium-CMC hydrogel was 15% higher than that of the sorbitan organogel. These data suggest that the mixing of two systems with similar viscosities would be an easier process without high mechanical loads. Although all the mechanical properties were about two times higher for hydrogels than for organogels, the hydrogel was characterized by better spreadability and the absence of the stickiness and oiliness characteristics typical of organogels. A higher consistency corelates with higher firmness, which can be explained by the formation of a spatial three-dimensional network structure of gellificator molecules [66] and better cohesion between particles. This, in turn ensures a uniform distribution of the gel on the skin without significant destruction of the gel structure. The results demonstrate that the different parameters of hydrophilic and lipophilic gels are determined by the bases and excipients of different origin. According to the scientific data, the type, and final properties of bigel systems are significantly influenced by the quantitative content of the oil and water phases [38,67,68].

### 3.2. Quality Evaluation for Prepared Bigels

**Figure 5:**
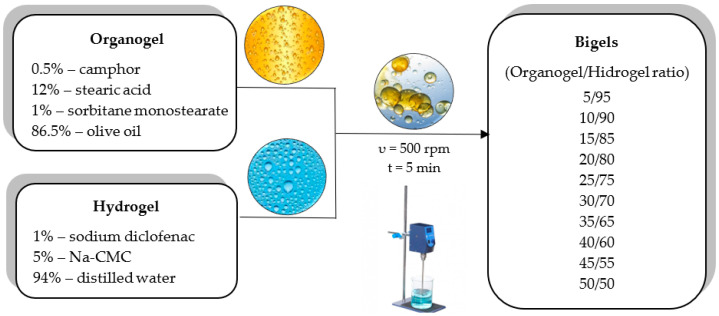
A schematic modelling of bigels with different ratios of hydrogel and organogel.

The modelling of the two-phase system carrier consisted of the following ratios of organogel/hydrogel, as shown in Figure 5: 5/95, 10/90, 15/85, 20/80, 25/75, 30/70, 35/65, 40/60, 45/55, and 50/50. 

The prepared samples were evaluated for their initial quality parameters: sensory properties, uniformity, stability, pH value, viscosity and textural properties.

#### 3.2.1. Effects of Organogel/Hydrogel Ratio on Sensory and Physical Properties of Bigels

The sensory and physical properties of bigels with different organogel/hydrogel ratios are presented in Table 4. 

Sensory evaluation of the prepared bigels found that they were homogenous semi-solid systems with colour depending on the amount of organogel. Olive oil has specific characteristics which influence the common sensory profile of products. All the samples had a camphor odour. 

The centrifugation test for biphasic systems showed that the bigels with a 45/55 and 50/50 ratio of constituents were unstable. Therefore, these two ratios were not further investigated. In the other samples, no phase separation was observed, indicating that organogel contents from 5 to 40% formed stable bigel systems.

The pH values of all the samples were in the range from 6.26 to 6.5, which corresponds to the physiological pH of the skin (4–6.5). These results indicate that the prepared bigels are suitable for topical application as they do not damage the skin’s protective barrier and cause irritation.

The viscosity results of the bigels with different constituent ratios (Table 4) demonstrate that viscosity depends on the amount of the lipophilic phase in the system (r = 0.97). With an increase in the amount of organogel from 5% to 40%, the dynamic viscosity increased 2.5 times (from 44,502 to 112,754 mPa·s). The increase in bigel viscosity with increasing organogel fraction was also observed by other researchers [38,39,40,44,53,68]. However, it should be noted that high viscosity can negatively affect the mechanical properties of the bigel, such as spreadability, as well as the skin feel and permeation of the incorporated active ingredients [69].

#### 3.2.2. Effects of Organogel/Hydrogel Ratio on Mechanical Properties of Bigels

The mechanical properties of bigels containing different ratios of organogel and hydrogel were studied by textural analysis using spreadability and back extrusion tests. 

**Figure 6:**
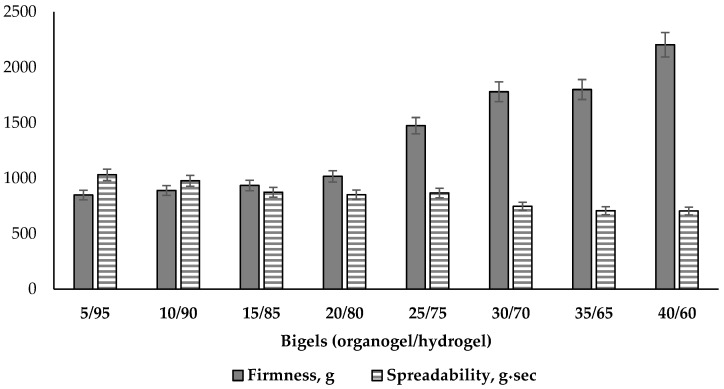
Firmness and spreadability parameters of bigel samples using spreadability test (n = 3).

The instrumental spreadability tests demonstrate the potential distribution on the skin, and this property also determines the extrudability from the package, the ease of application, the accuracy of dosing, and suitability for patient preferences [38,68,70]. The results of the tests for firmness and spreadability are presented in Figure 6. 

According to the results in Figure 6, the force of deformation, which is used to determinate the firmness of bigels, increased 2.6 times (from 849 to 2204 g). This variation is correlated with increasing organogel content: a very strong correlation (r = 0.95) was found between the amount of organogel in the two-phase system and the value of the firmness parameter, as well as between firmness and viscosity (r = 0.91). The first three samples of bigels had the lowest similar firmness with amounts of organogel from 5 to 15%, and there was no statistically significant difference between them (*p* ≥ 0.05). The results of the bigel systems with the phase ratios of 25/75, 30/70, 35/65 were higher than the others and increased significantly. The sample with the highest organogel content, 40/60, had the highest firmness. Therefore, the obtained results indicate that the firmness and the viscosity of the bigels directly depend on the amount of organogel in the two-phase system. Similar conclusions were also reached by Vinay K. Singh and co-authors: increasing organogel in hydrogel base influences the firmness of bigel [39].

Viscosity is the main factor impacting spreadability of semi-solid dosage forms [71]. Increased viscosity indicates that the bigel texture is becoming firmer, so it may be more difficult to spread it on the skin. This is also confirmed by the decrease in spreadability value with increasing organogel content in the system (Figure 6). A very strong correlation was also found between the spreadability and firmness values (r = 0.89)—as the bigel firmness increased, spreadability decreased. The highest spreadability (about 1000 g·s) was determined in the first two samples with 5/95 and 10/90 ratios (*p* ≥ 0.05). The medium values of this parameter are typical for systems with ratios of 15/85, 20/80, 25/75, and they showed statistically significant differences compared to other results. The lowest spreadability results were determined in the 30/70, 35/65 and 40/60 systems (*p* ≤ 0.05)—these bigels were the most difficult to apply and spread over the skin compared to other tested samples. Agarwal M. and co-authors also concluded that the amount of organogel determines the formulation’s consistency and viscosity: their formulation with the lowest viscosity showed good spreadability, and a formulation with the highest viscosity showed the lowest spreadability [72].

It is also necessary to pay attention to the fact that the difference between the “firmness-spreadability” indicators of the samples increases with the rise in the proportion of the oily phase in the system. Thus, in samples with proportions of organogel from 5% to 20%, the studied indicators differ from each other by no more than 1.2 times, and samples with an organogel content of 25–40% differ in their values of “hardness” and “flowability” by 1.7–3.1 times.

**Figure 7:**
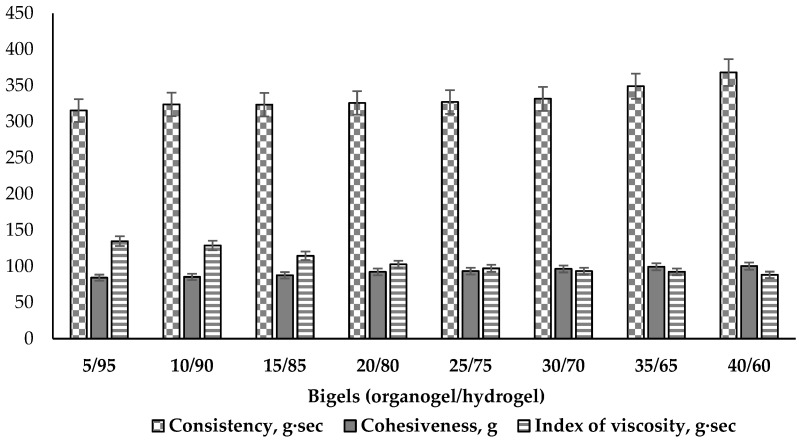
Consistency, cohesiveness and index of viscosity of bigel samples using back extrusion test (n = 3).

Other mechanical properties of bigels—consistency, cohesiveness and index of viscosity—were determined performing the back extrusion test. Consistency describes the fluidity and degree of firmness of a viscous plastic material. Cohesiveness describes the strength of the internal structural bonds of bigel phases, which through the interaction of molecules, enable the bigel to keep a stable shape. This parameter shows the simulating force required to extrude the sample and describes the value of deformation before rupture [73]. The index of viscosity demonstrates the work of adhesion. The results are given in Figure 7.

The results of the consistency tests demonstrate a small (about 16%) increase related to higher amounts of the oily phase in the bigels (Figure 7). The higher consistency values tend to result in the sample having a thicker and harder form. From a biopharmaceutical perspective, products with a low consistency can be more thoroughly applied on the skin and more easily absorbed [74]. The lowest consistency was determined in the 5/95 sample (315.54 g·s), and the bigels with ratios 10/90, 15/85, 20/80, 25/75, 30/70 had similar values (324.03–331.69 g·s). Statistically significant differences were observed beginning with the 35/65 ratio bigel (35/65—349.06 g·s; 40/60—368.05 g·s). This change in consistency parameters make this a meaningful composition ratio among the analysed bigels. This direct correlation between the firmness and consistency of the bigel has also been established by A. Martin-Illana and co-authors [75].

The cohesiveness of the bigel samples increased with a higher content of the organogel (Figure 7). This can be explained by the increase in the amount of emulsifier present in the organogel, which is able to reduce the interfacial tension between the oil and water phases and stabilize the system [76]. Comparing all the results of cohesiveness, it was noted that they split to three levels according the statistical significance. The lowest cohesiveness (84–87 g) was determined in the first three 5/95, 10/90 and 15/85 ratio systems (*p* > 0.05); medium levels (92–96 g) were observed for the 20/80, 25/75 and 30/70 ratios (*p* > 0.05), and the highest levels (99–100 g) were measured in the 35/65 and 40/60 ratio bigels (*p* > 0.05). This indicates the greater inner strength of these bigels and their better ability to withstand external forces without significant structural destruction. However, the bigel samples with the highest proportions of the lipophilic phase (35 and 40%) were characterized by a greasy feeling when applied to the skin, which may be unpleasant for the user. The other bigels (5/95, 10/90, 15/85, 20/80, 25/75) did not cause such a feeling.

Many authors have described index of viscosity as the extrusion energy or work of adhesion when it becomes higher [74,77,78]. In this study, it was observed that the higher the cohesiveness, the stronger the inner intermolecular bonds in the sample of the bigel and the lower the adhesion to the tool of the device. Less viscous systems, with less cohesiveness (5/95, 10/90, 15/85), in contrast, were characterized by higher adhesion, and therefore, a larger value of the index of viscosity; more resistance was encountered when pulling the analyzer tool from a bigel sample. This confirms the fact that viscosity is related to cohesiveness and consistency [73,79].

According to the results of the texture analysis, it can be concluded that the increase in organogel content in the composition of the bigel determines its mechanical properties. The high firmness systems (30/70, 35/65, 40/60) were characterized by the lowest spreadability value and the highest consistency; therefore, the topical application is likely to be uncomfortable. The less firm bigels (5/95, 10/90, 15/85) had low firmness and cohesiveness, medium consistency, and the highest adhesion to the appliance tool. These qualities, in turn, can negatively affect the consumer properties of the product. At this stage of the study, suitable mechanical properties were selected that would be acceptable to the consumer—medium firmness and cohesiveness, and good consistency and spreadability. Considering this, the bigels with 20/80 and 25/75 organogel/hydrogel ratios were selected for further study. 

#### 3.2.3. Effects of Organogel/Hydrogel Ratio on Rheological Properties of Bigels

The structural and mechanical properties of the bigel samples with the organogel/hydrogel ratios of 20/80 and 25/75 were also investigated. The rheological parameters were measured using a viscometer MCR102 (Anton Paar, Graz, Austria), equipped with a plate-plate geometry, at temperatures of 25 °C (room, storage temperature) and 32 °C (skin, application temperature). The results of the investigated bigels’ dynamic viscosity and shear rate during forward and reverse movement are presented in Table 5.

Analysis of the results given in Table 5 shows that for the investigated samples, the structural viscosity gradually decreases with an increase in the shear rate gradient. This dependence is typical for systems with a plastic type of flow and characterizes the studied bigels as structured dispersed systems, which will ensure their easy and uniform distribution on the skin [74,79]. The viscosity of both tested samples at storage (25 °C) and application (32 °C) temperatures changes slightly—no more than 1.2 times. This indicates a certain thermal stability of the drug in the required temperature range. In addition, there is no statistical difference between the viscosity values of the two bigel samples.

**Figure 8:**
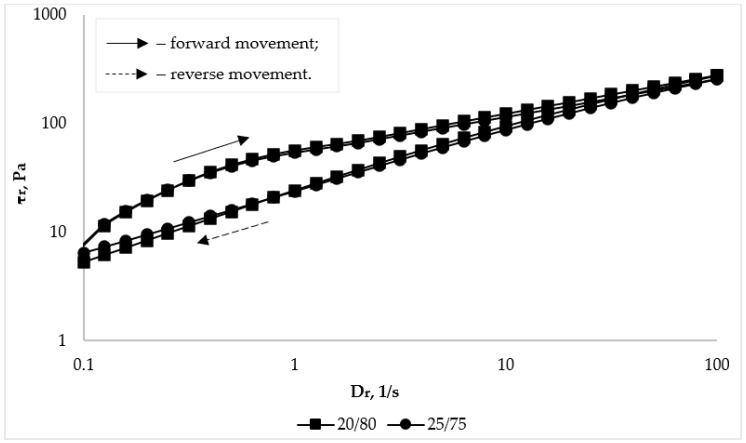
Dependence of shear stress (τr) on shear rate (Dr) of bigel samples with different organogel/hydrogel ratios at a temperature of 25 °C.

The non-Newtonian pseudoplastic behavior of flow of the studied bigel samples is also evidenced by the obtained rheograms (Figure 8).

This conclusion is confirmed by the fact that the rheological curves in the studied range of shear stresses are well described by the Ostwald-de Waele exponential equation:

τ = Kγ^n^,

where τ is the shear stress (Pa), K is the consistency coefficent (Pa·s^n^), γ is the shear rate (s^−1^), and n is the flow behavior index (for structured systems with pseudoplastic behavior of flow n < 1, for Newtonian fluids n = 1) [79]. The thermorheological characteristics of the bigel samples at temperatures of 25 °C and 32 °C are given in Table 6.

According to the data presented in Table 6, the n value is less than 1, which indicates the plastic behavior of flow of the studied samples. There was no difference in the samples’ rheoparameters with the increase in the proportion of organogel in the biphasic system from 20% to 25%. In addition, no statistically significant differences were found between the rheoparameters when the temperature increased, which indicates the thermal stability of the investigated bigel systems.

The thixotropy of the system is evidenced by the dependences of viscosity or shear stress, which characterize the behavior of the system during reverse movement, i.e., shear rates from 100 s^−1^ to 0.01 s^−1^, which are located below the curves during forward movement. The curves of the reverse course being located above the curves of the forward course would indicate the rheopectic behavior of the system [80]. As can be seen from the data in Figure 8 and Table 5, the reverse movement curves are located below the forward movement curves. This indicates that the investigated bigels are characterized by thixotropic behavior, that is, the bigels are able to restore their structure after removal of external forces (load).

#### 3.2.4. Effects of Organogel/Hydrogel Ratio on Particle Size and Distribution in Bigels

Particle size and distribution play an important role in formulations used for topical or transdermal drug delivery, as these parameters affect the stability, release, permeation, and bioavailability of the drug [74,81,82]. 

**Figure 9:**
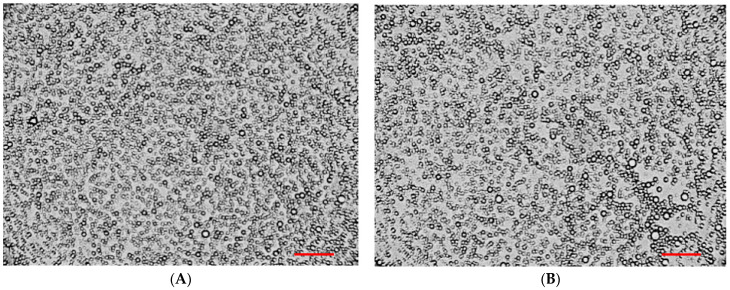
Microscopic analysis of bigel samples: (**A**)—20/80, (**B**)—25/75. Reference bar corresponds to 100 μm.

To assess the particle size and their distribution in the bigels, a microscopic analysis of selected samples was carried out. Optical microscopy results at 100× magnification are shown in Figure 9.

The results in Figure 9 suggest that an oil-in-water pattern of bigels is produced. The bigel with organogel/hydrogel ratio 20/80 demonstrated a more uniform distribution of oil-phase spherical particles in the system; their sizes varied in the range 0.1–10 μm (Figure 9A). In the sample with a ratio of 25/75, the observed particle size was also in the specified range; however, in the visible region, cases of droplets sticking and uneven distribution were noted (Figure 9B), which may be associated with the higher content of organogel in the composition of the biphasic system. 

Similar conclusions were drawn by other researchers using various microscopic methods in the study of different compositions of bigels: V. Andonova with co-authors [68], F.R. Lupi et al. [34], A. Mazurkeviciute and co-authors [41], and A.J. Martins et al. [67] also noted that a larger particle size and more heterogeneous non-uniform microstructures were observed when the organogel proportion in the bigel formulation was increased. 

**Figure 10:**
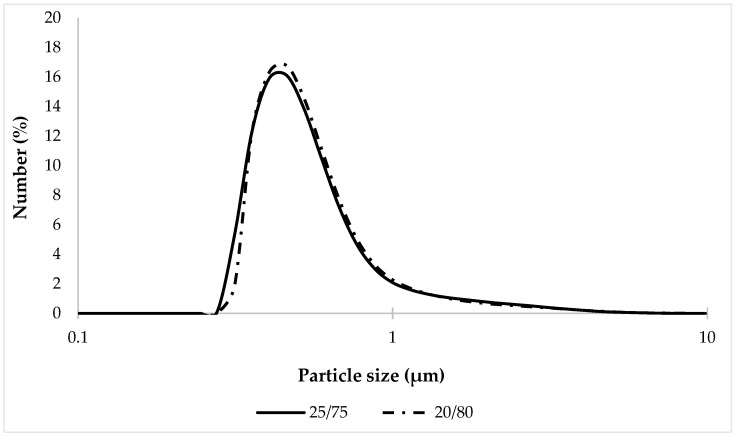
Particle size distribution of tested bigel samples.

Since microscopic analysis provides only a superficial understanding of system dispersity, it was decided to assess the uniformity of particle size distribution in the tested samples using the laser diffraction technique. The obtained results of particle size distribution are shown in Figure 10.

The results of the analysis (Figure 10) showed that the percentage content of the oil phase in the samples 20/80 and 25/75 has a slight effect on the size and distribution of particles; no statistically significant differences between the studied compositions were found.

When comparing percentiles D10, D50, and D90 (Table 7), it was established that the particle size distribution of the tested samples was in the same range: 20/80—0.388–1.04 μm; and 25/75—0.374–1.05 μm. This may be a consequence of the two-phase structure formed by the simultaneous combination of an emulsifier and a rheology modifier (thickener), which improves the kinetic stability of the system and thus limits the movement, aggregation and coalescence of droplets [83]. It should also be noted that the maximum particle size, the content of which was very small (0.01%), was 7.64 µm for the 20/80 sample and 8.68 µm for the 25/75 sample, which confirms the results of the microscopic analysis.

#### 3.2.5. Effects of Organogel/Hydrogel Ratio on Dynamics of API Release

**Figure 11:**
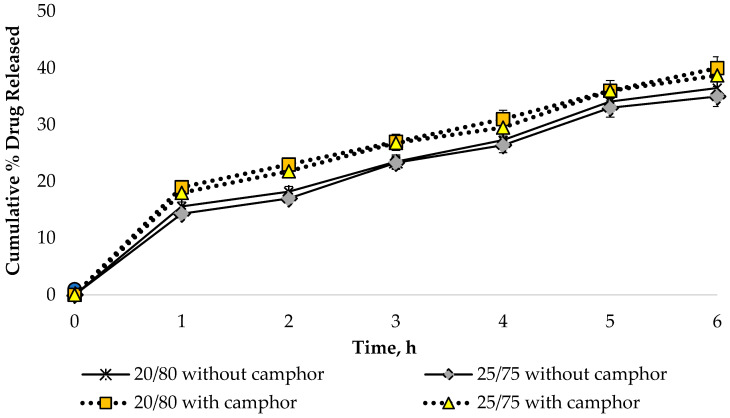
Dynamics of sodium diclofenac release from bigel samples (n = 3).

In vitro release studies are an important step in the development of pharmaceutical products since the bioavailability of APIs is affected by the chosen dosage form and excipients. An in vitro release assay was performed to determine the release dynamics of sodium diclofenac from the bigel samples in the presence and absence of camphor using the method of dialysis through a semi-permeable membrane. The HPLC technique was used to quantify sodium diclofenac. Dialysate samples were taken at 0.5, 1, 2, 3, 4 and 6 h after the start of the experiment. In the calculations, 1.0 g of diclofenac sodium was considered as a 100% result, representing its content in the formulation under development. The obtained data are shown in Figure 11.

According to the data presented in Figure 11, a uniform increase in the amount of API in the dialysate was observed during the 6 h study, which indicates a prolonged release of sodium diclofenac from the bigel samples. 

The release of sodium diclofenac was found to be significantly higher in the bigel samples containing camphor (*p* ≤ 0.05). However, there was no significant difference in the extent of API release between samples with ratios of 20/80 and 25/75. This indicates that variations in the amount of organogel within the range of 20–25% had no effect on the release of sodium diclofenac from the bigel. The design of a combination of lipophilic and hydrophilic properties can be projected to ensure the successful release and permeation of the active substance into human skin in topical dosage forms. This experimental finding is supported by other researchers. Pradal J. and co-authors also proved the importance of the composition and dosage form for local administration of diclofenac through the skin. In their studies, they demonstrated significantly higher skin permeation of diclofenac-containing emulsion compared to diclofenac sodium gel [84,85]. Manian M. with co-authors evaluated emulsion, emulgel, gel, and ointment compositions containing diclofenac sodium and established a significantly higher degree of drug permeation and retention for the emulgel composition [86]. 

### 3.3. Quantitative Analysis of APIs in the Bigel Formulation

**Figure 12:**
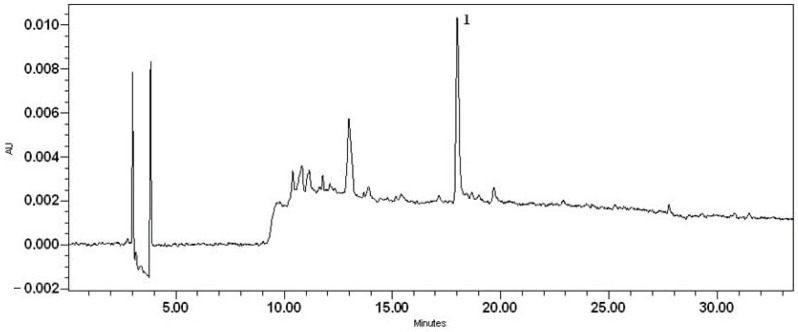
The chromatogram of sodium diclofenac bigel sample (1—sodium diclofenac).

An important step in the development of a new dosage form is the quantitative determination of the APIs. Quantification of sodium diclofenac was determined by High-Pressure Liquid Chromatography—Photodiode Array Analysis. The concentration of sodium diclofenac in the bigel is calculated from a linear regression equation y = 2.10 × 10^6^x − 2.39 × 10^4^, where y corresponds to the peak area and x to the substance concentration (Figure 12). The concentration of sodium diclofenac in the bigel sample was determined to be 1% with an 0.002% accuracy. Each sample was analysed in triplicate and the mean value was used for calculation. The concentration of sodium diclofenac was expressed as mg/mL of the bigel, then converted to percentage concentration. The obtained concentration of sodium diclofenac corresponds to the amount added during the formulation of the bigel.

**Figure 13:**
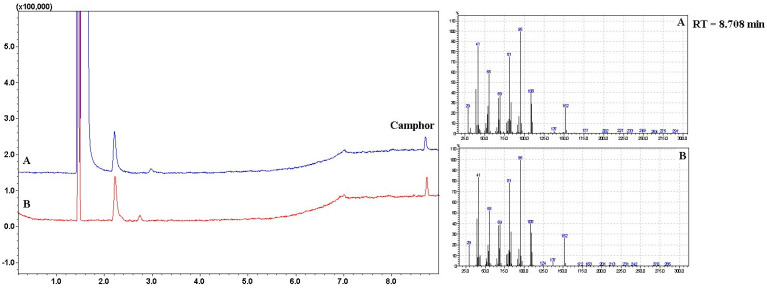
The GC-MS chromatogram of camphor bigel samples (A—bigel sample, B—camphor standard solution).

Quantitative determination of camphor was carried out by gas chromatography, which is the preferred method for the analysis of volatile substances. This method of determining camphor has also been used by other authors in the analysis of different preparations [23,29,30]. Identification of camphor by Gas Chromatography-Mass Spectrometry Headspace Analysis was carried out using mass spectra library search (NIST 14) and compared with the mass spectral data from the literature and a standard solution [29]. The quantification of camphor was performed by comparison with the standard solution peak area. The concentration of camphor in the bigel was determined to be 0.5% with an 0.002% accuracy. The obtained concentration of camphor corresponds to the amount of camphor added during the formulation of the bigel. Figure 13 shows the GC-MS chromatograms obtained in the GC-MS headspace investigation.

### 3.4. Stability Evaluation of Bigels

Considering that two-phase systems are prone to destabilization, bigels are usually subjected to stability studies at various temperatures and humidity values. Therefore, we decided in our further research to determine of the storage stability of the two bigel samples (20/80 and 25/75) with diclofenac sodium and camphor. The developed products were stored under two different sets of conditions: in a climatic chamber (40 ± 2 °C/75 ± 5% RH) and at room temperature (25 ± 2 °C/60 ± 5% RH) [34,72,87,88]. Quality parameters (sensory properties, uniformity, pH value, centrifugation and freeze–thaw stability tests, viscous and mechanical characteristics, APIs content) were evaluated during 6 months of storage according to the ICH Guidelines Stability [39,54,87,89,90].

The organoleptic properties of the bigel samples and the pH values remained unchanged during storage under the two temperature regimes. The samples passed the colloidal and freeze–thaw stability tests, and no phase separation or other changes were observed, which confirms the resistance of the two-phase system to temperature influences and their stability over all the test intervals. During the control period of storage, the quantitative content of APIs was also determined, which remained within the permissible limit of 5 percent. The structural-mechanical and viscosity characteristics of the bigels also did not undergo significant changes. Only under the condition of 40 ± 2 °C/75 ± 5% RH were slight variations in mechanical properties observed, but no phase separation was observed.

Therefore, based on the conducted research, it was established that throughout the entire storage period under two temperature regimes, the developed bigels with camphor and diclofenac sodium remained physically, chemically, and technologically stable for 1, 3, and 6 months. V. K. Singh and co-authors also established the bigels’ stability at room temperature for 6–12 months [54].

## 4. Conclusions

In this research, bigels containing a combination of sodium diclofenac and camphor as APIs for the treatment of muscle-joint inflammation and pain was investigated for the first time. 

This study presents the development and evaluation of improved semi-solid two-phase formulations for topical use based on technological parameters. The ratio of organogel and hydrogel had the greatest influence on the quality parameters. 

Rheological and textural analysis established increases in bigel viscosity, firmness, and cohesiveness with higher percentages of organogel. However, as the firmness (viscosity) of the two-phase system increases, the spreadability indicator decreases. Thus, the high-firmness systems (30/70, 35/65, 40/60) were characterized by the lowest spreadability values and the highest consistency, meaning that their topical application would not be comfortable. The less firm bigels (5/95, 10/90, 15/85) showed low firmness and cohesiveness, medium consistency, and the highest adhesion to the appliance tool. These qualities, in turn, can negatively affect the sensory profile of the product. Medium firmness and cohesiveness, good consistency and spreadability that would be acceptable to the consumer, were characteristic of bigels with ratios 20/80 and 25/75. These bigel ratios were identified as suitable carriers for sodium diclofenac and camphor, which was evaluated and confirmed by the results of the rheological and microscopic analysis. The quantity of sodium diclofenac and camphor were determined by HPLC and gas chromatography. 

The combination of these two gels exhibited a synergistic effect, leading to improved drug release attributed to the dual hydrophilic and lipophilic properties of the carriers, as demonstrated by in vitro studies. Future research efforts will focus on elucidating bigel bioavailability to further characterize the release and permeation of active ingredients using human skin. Additionally, ongoing stability studies will be extended to assess the formulations over a longer period. 

## Figures and Tables

**Table 1:** Excipients used in bigels.

Hydrogels	Organogels
Solvent: purified water Hydrophilic gelling agents: - synthetic polymers (carbopol [38,39], polyvinyl alcohol [40], polyvinylpyrrolidone [40], sodium polyacrylate, acrylamide, poloxamer 407 [41]); - semi-synthetic polymers (hydroxypropyl methylcellulose [33,42,43,44], hydroxyethyl cellulose, sodium carboxymethylcellulose [45]); - natural polymers (alginates [42], gelatine [46,47,48], collagen [43], starch [45,49], agarose [47,50], pectin [34,44], chitosan [44], silicon dioxide [51], hyaluronic acid, carrageenan [52], locust bean gum, guar, arabic and xanthan gums [53,54,55]).	Solvent: - organic solvents: benzene, hexane [56]; - vegetable oils: sunflower [40,48,55], castor [56], peanut, olive [34,49], sesame [43,44,46], soybean [46], almond [38], corn [52], pomegranate seed oil [57], tea tree oil, fish oil [42,58]. Organogelators/emulgators: - sorbitan esters: sorbitan monostearate (Span 60) [33,38,39,44,54], sorbitan monopalmitate (Span 40) [48,55,56], sorbitan monooleate (Span 80) [53]; - waxes: beeswax, propolis and candelilla [57,59,60]; - soy lecithin [33]; - ethylcellulose [61]; - colloidal silicon dioxide [51]; - fatty acids and alcohols: cetyl stearyl alcohol [33,50,61], stearic acid [46,62], 12-hydroxystearic acid [63].

**Table 2:** Analytical characteristics of the HPLC method validation data.

Characteristics	Data
Average Retention Time, RT	17.79
Relative standard deviation, RSD	0.001
Accuracy, %	0.002
Confidence Interval of RT, (min)	17.78–17.80
Limit of detection, LOD (µg/mL)	0.434
Limit of quantitation, LOQ (µg/mL)	1.449
Regression equation	y = 2.10 × 10^6^x − 2.39 × 10^4^
Correlation coefficient, R^2^	0.9987

**Table 3:** Physical and mechanical properties of organogel and hydrogel.

Parameter	Organogel	Hydrogel
pH	6.25 ± 0.31	6.45 ± 0.32
Viscosity (mPa·s)	64,745 ± 3237	76,213 ± 3810
Firmness (g)	130.6 ± 6.53	329.17 ± 16.45
Spreadability (g·s)	87.85 ± 4.3	201.69 ± 10.08
Consistency (g·s)	287.72 ± 14.38	419.26 ± 20.96
Cohesion (g)	160 ± 8.10	210 ± 10.50
Viscosity index (g·s)	18.64 ± 0.9	28.26 ± 1.41

Note. n = 3, *p* < 0.05.

**Table 4:** Sensory and physical properties of bigels.

Bigels (Organogel/Hydrogel Ratio)	Odour	Colour	Uniformity	Stability (after Centrifugation)	pH	Viscosity, mPa·s
5/95	Light CO	White	H	+	6.26 ± 0.3	44,502 ± 222
10/90	Light CO	White	H	+	6.43 ± 0.3	52,242 ± 261
15/85	CO	White	H	+	6.50 ± 0.3	71,750 ± 358
20/80	CO	White	H	+	6.41 ± 0.3	76,502 ± 382
25/75	CO	White	H	+	6.35 ± 0.3	80,594 ± 402
30/70	CO	Yellowish	H	+	6.40 ± 0.3	87,090 ± 435
35/65	Intense CO	Yellowish	H	+	6.50 ± 0.3	90,848 ± 454
40/60	Intense CO	Yellowish	H	+	6.48 ± 0.3	112,754 ± 563
45/55	Intense CO	Yellow–creamy	NH	—	-	-
50/50	Intense CO	Yellow–creamy	NH	—	-	-

Notes. n = 3, *p* < 0.05, “-”—not measured because bigel systems separated after centrifugation; “CO”—camphor odour; “H”—homogeneous, “NH”—non-homogeneous; “+”—stable (no phase separation); “—”—unstable (phase separation).

**Table 5:** Dependence of the dynamic viscosity of bigels with different organogel/hydrogel ratios on temperature.

D_r_, 1/s	Viscosity (η), mPa·s			
	20/80		25/75	
	25 °C	32 °C	25 °C	32 °C
0.199	97,193	86,763	98,758	92,001
1	56,712	48,709	53,790	48,321
2	34,977	29,657	33,153	29,643
5.01	19,280	16,305	18,115	15,889
10	12,347	10,431	11,449	10,018
20	7901.9	6653.7	7265.3	6342.6
50.1	4379.9	3675.9	3991.9	3460.2
100	2797.3	2384.2	2561.6	2202.6
100	2775.9	2354.5	2559.6	2179.6
50.1	4138.7	3398.3	3803.8	3077.9
20	6768.3	5450.5	6198.4	4756.7
10	9487.2	7521.6	8718.2	6389.9
5.01	12,937	10,160	11,991	8694.3
2	18,788	14,694	17,811	13,529
1	24,248	19,179	23,771	18,485
0.199	41,918	35,464	47,563	37,136

**Table 6:** Thermorheological characteristics of bigel samples (n = 3).

Parameters	Organogel/Hydrogel Ratio			
	20/80		25/75	
	25 °C	32 °C	25 °C	32 °C
Coefficient of consistency (*K*)	42.932 ± 1.361	39.652 ± 1.112	46.119 ± 1.580	39.722 ± 1.254
Flow index (*n*)	0.452 ± 0.005	0.423 ± 0.003	0.424 ± 0.002	0.424 ± 0.002
Coefficient of determination (*R*^2^)	0.9476	0.9612	0.9816	0.9802

**Table 7:** Particle size distribution of the studied bigels (n = 5).

Sample	D10 (µm)	D50 (µm)	D90 (µm)
20/80	0.388 ± 0.022	0.536 ± 0.019	1.040 ± 0.031
25/75	0.374 ± 0.017	0.524 ± 0.028	1.050 ± 0.025

## Data Availability

All data are available upon request.
